# Data-driven discovery and validation of circulating blood-based biomarkers associated with prevalent atrial fibrillation

**DOI:** 10.1093/eurheartj/ehy815

**Published:** 2019-01-07

**Authors:** Winnie Chua, Yanish Purmah, Victor R Cardoso, Georgios V Gkoutos, Samantha P Tull, Georgiana Neculau, Mark R Thomas, Dipak Kotecha, Gregory Y H Lip, Paulus Kirchhof, Larissa Fabritz

**Affiliations:** 1 Institute of Cardiovascular Sciences, University of Birmingham, Birmingham, UK; 2 Institute of Cancer and Genomic Sciences, University of Birmingham, Birmingham, UK; 3 Department of Cardiology, Sandwell and West Birmingham Hospitals NHS Trust, Birmingham, UK; 4 Department of Cardiology, University Hospitals Birmingham NHS Foundation Trust, Birmingham, UK

**Keywords:** Atrial fibrillation, Biomarkers, Machine learning, BNP, FGF-23, Validation

## Abstract

**Aims:**

Undetected atrial fibrillation (AF) is a major health concern. Blood biomarkers associated with AF could simplify patient selection for screening and further inform ongoing research towards stratified prevention and treatment of AF.

**Methods and results:**

Forty common cardiovascular biomarkers were quantified in 638 consecutive patients referred to hospital [mean ± standard deviation age 70 ± 12 years, 398 (62%) male, 294 (46%) with AF] with known AF or ≥2 CHA_2_DS_2_-VASc risk factors. Paroxysmal or silent AF was ruled out by 7-day ECG monitoring. Logistic regression with forward selection and machine learning algorithms were used to determine clinical risk factors, imaging parameters, and biomarkers associated with AF. Atrial fibrillation was significantly associated with age [bootstrapped odds ratio (OR) per year = 1.060, 95% confidence interval (1.04–1.10); *P *=* *0.001], male sex [OR = 2.022 (1.28–3.56); *P *=* *0.008], body mass index [BMI, OR per unit = 1.060 (1.02–1.12); *P *=* *0.003], elevated brain natriuretic peptide [BNP, OR per fold change = 1.293 (1.11–1.63); *P *=* *0.002], elevated fibroblast growth factor-23 [FGF-23, OR = 1.667 (1.36–2.34); *P *=* *0.001], and reduced TNF-related apoptosis-induced ligand-receptor 2 [TRAIL-R2, OR = 0.242 (0.14–0.32); *P* = 0.001], but not other biomarkers. Biomarkers improved the prediction of AF compared with clinical risk factors alone (net reclassification improvement = 0.178; *P *<* *0.001). Both logistic regression and machine learning predicted AF well during validation [area under the receiver-operator curve = 0.684 (0.62–0.75) and 0.697 (0.63–0.76), respectively].

**Conclusion:**

Three simple clinical risk factors (age, sex, and BMI) and two biomarkers (elevated BNP and elevated FGF-23) identify patients with AF. Further research is warranted to elucidate FGF-23 dependent mechanisms of AF.

**Figure:**
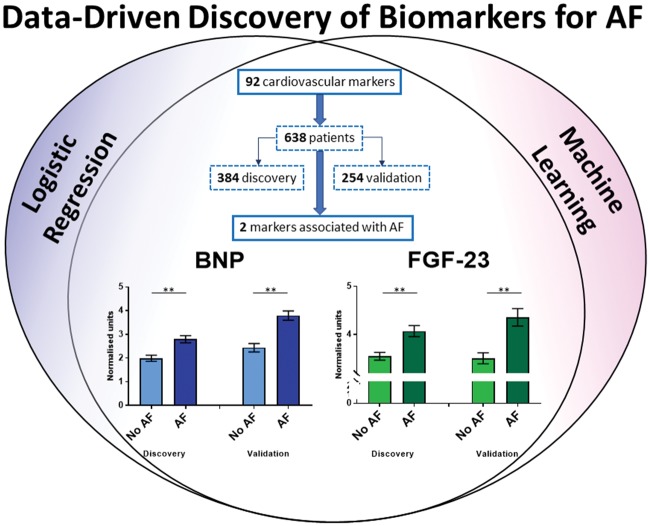

## Introduction

Atrial fibrillation (AF) is often only identified after a complication, e.g. a stroke.^1^^,^^2^ Initiation of oral anticoagulation can prevent such events,^3–5^ leading to calls for systematic AF screening in at risk populations^6^ to allow timely initiation of anticoagulation. Unfortunately, ECG screening is resource-intensive and burdensome for patients.^7^ Therefore, clinical risk factors associated with AF^8^ such as older age, prior stroke, obesity, hypertension, diabetes, ischaemic heart disease, chronic kidney disease, and heart failure, are used to identify subpopulations suitable for ECG screening. These risk factors, individually or in combination, have modest predictive ability and their determination requires specialist knowledge (e.g. for diagnosing heart failure), presenting a challenge for effective screening.

Blood biomarkers have the potential to support community screening programmes for AF (e.g. incorporated into point-of-care tests). Several candidate biomarkers for detection of AF have been proposed, such as N-terminal prohormone of brain natriuretic peptide (NT-proBNP)^9^ and brain natriuretic peptide (BNP)^10^ reflecting atrial strain, C-reactive protein^11^ reflecting inflammation, Galectin-3^12^ correlating with cardiac fibrosis, or cystatin or glomerular filtration rate^13^ as a marker of chronic kidney disease. Brain natriuretic peptide, the best-studied marker, is similarly elevated both in patients with prevalent AF^10^^,^^14^ and in cohorts analysed for incident AF.^9^^,^^15^ So far, most analyses identifying biomarkers in patients with AF have been hypothesis-driven and involved measurement of a single or a small selection of blood biomarkers.^16^ These biomarkers also compete with other cardiovascular markers related to prognosis or diagnosis of other cardiac conditions (e.g. heart failure, atherosclerosis, and coronary events) or death.^16^

To enable a data-driven analysis of AF specific biomarkers, we quantified 40 cardiovascular biomarkers in an unselected cohort of patients. All patients without known AF were screened for silent, undiagnosed AF using 7-day event monitoring. We combined biomarker concentrations with known clinical risk factors of AF to determine which markers best distinguish patients with and without AF. In a secondary analysis, we also included imaging parameters that have been associated with AF.^17^ Using both logistic regression and machine learning algorithms, we identified robust markers for AF.

## Methods

### Study population

Seven hundred and twenty consecutive patients referred to the Sandwell and West Birmingham Hospitals NHS Trust (Birmingham, UK), for inpatient or outpatient evaluation of acute illnesses were recruited between September 2014 and August 2016 as part of the Birmingham and Black Country Atrial Fibrillation Registry (BBC-AF). Eligible patients either had diagnosed AF (confirmed by ECG^4^) or at least two CHA_2_DS_2_-VASc stroke risk factors.^4^ Complete enrolment criteria are given in Supplementary material online, *Table S1*. All patients without diagnosed AF underwent 7-day ambulatory ECG monitoring to detect silent AF. Clinical information was obtained from a detailed interview, review of electronic patient records, and chart review. Transthoracic echocardiography was performed in all patients. For analysis purposes, the cohort was divided chronologically in an approximate 60:40 ratio, conventional for discovery-validation paradigms (discovery cohort: patients 1–450; validation cohort: patients 451–720). This study complied with the Declaration of Helsinki, was approved by the National Research Ethics Service Committee (BBC-AF Registry, West Midlands, UK, IRAS ID 97753), and was sponsored by the University of Birmingham, UK. All patients provided written informed consent.

### Biomarker quantification

Blood samples from all patients were fractionated and stored at −80°C until analysis. Protein concentrations were quantified with standardized methods using a validated proximity extension assay which simultaneously measured all protein concentrations from 1 µL of EDTA plasma (Olink Proteomics, Uppsala, Sweden; for details see Supplementary material online, *Methods*). Data from 82 patients (11%) were removed due to assay failure and/or flagging during quality control, and excluded from analysis. These patients were not different in clinical characteristics from the rest of the cohort. All data were analysed as log-2 transformed units (fold change). For technical reasons (supply of the panel kits), Olink cardiovascular Panel I was used in the discovery cohort, and Olink cardiovascular Panel II in the validation cohort. Out of the 92 proteins quantified on each panel, 52 were unique to either panel. The remaining 40 overlapping proteins between the two panels were included in the primary analysis (Supplementary material online, *Table S2*).

### Statistical analysis

The baseline characteristics of patients with and without AF in both the discovery and validation cohorts were compared. Categorical variables were assessed using χ^2^ tests. Continuous variables were compared using independent samples *t*-tests or Mann-Whitney *U* tests as applicable after testing for data normality with the Kolmogorov–Smirnov test. A two-tailed *P*-value of <0.05 was considered to be statistically significant.

**Figure 1:**
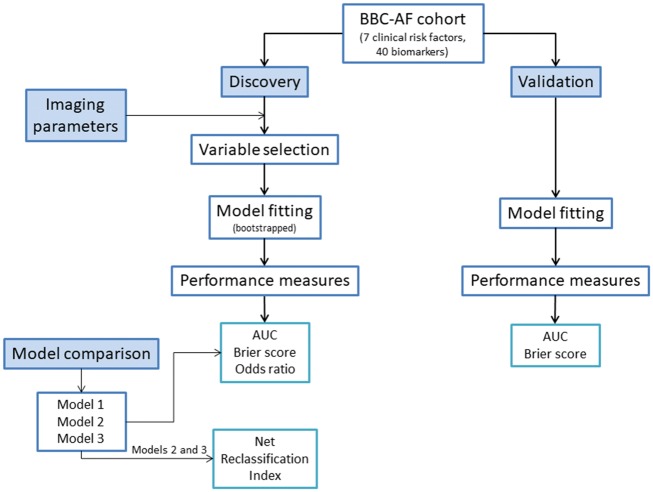
Forward selection logistic regression methods. Primary analysis involved forward selection for identifying variables to be fitted in the logistic regression model for biomarker discovery, evaluation of model performance in the validation cohort, comparison of performance measures with two other models, and quantification of net reclassification index, as well as inclusion of imaging parameters in the secondary analysis.

Using data from the discovery cohort, we considered all 40 biomarkers and seven clinical risk factors [age, sex, hypertension, heart failure, history of stroke or transient ischaemic attack, kidney function, and body mass index (BMI)] for variable selection. Values missing at random were imputed (see Supplementary material online, *Methods* for details). Forward selection with an entry criterion of *P *=* *0.05 was applied as an objective, data-driven technique to identify the smallest number of variables required for a practical model. Using logistic regression, the selected biomarkers and clinical risk factors were modelled for their association with AF in the discovery cohort, and subsequently evaluated in the validation cohort (*Figure 1*). Bootstrapping was used to adjust model coefficients for over-optimism due to potential over-fitting in the discovery data. The area under the receiver-operator curve (AUC or C-statistic) and Brier score were calculated using SPSS v.24 (IBM Corporation, Armonk, NY, USA). In a sensitivity analysis, all 92 biomarkers available in the first 450 patients were included for variable selection and modelling (Supplementary material online, *Table S3*). In an additional sensitivity analysis, we randomly allocated patients to the discovery and validation cohorts using random case sampling instead of splitting the cohort by biomarker panel (Supplementary material online, *Analysis A1*).

We further assessed the AUC of two additional models, the first with only age as the predictor,^18^ the second with only clinical risk factors selected in the forward selection procedure, and compared the AUCs with the model which included biomarkers. The net reclassification index (NRI) was calculated to assess the added discriminative ability of biomarkers, using Matlab 2017a (The MathWorks, Inc., Natick, MA, USA). In a secondary analysis, we evaluated the impact of including two imaging parameters that have been associated with AF (mitral valve disease and left atrial dilation)^19^ on the selection of biomarkers. Presence of mitral regurgitation was equivalent to Grade II and above, whereas the left atrial dilation was defined as mild, moderate, or severe dilation on echocardiography.

### Machine learning

**Figure 2:**
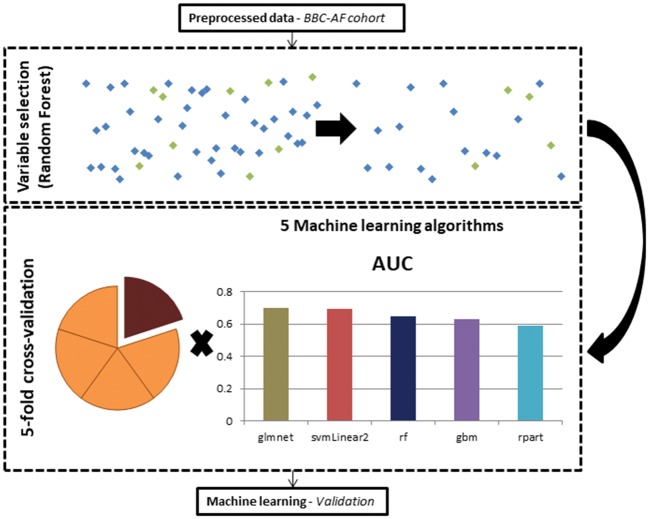
Machine learning methods. Analysis involved feature selection using the Random Forest algorithm, and model training using five different algorithms and five-fold cross-validation.

Using the imputed dataset, each continuous variable was centralized to the mean and scaled to the standard deviation, whereas categorical variables were coded into binary numbers (0 and 1). Backward feature selection with the random forest algorithm was used to identify variables for inclusion in the model according to the best AUC. Models were then created using five-fold cross-validation with each training unit being sampled using random over-sampling examples (*Figure 2*). The best model from cross-validation was trained using the whole discovery dataset and evaluated on the validation dataset. The performance of machine learning models was also evaluated by the AUC (see Supplementary material online, *Methods* for details). The R language for statistical computing was used for analysis.

## Results

### Elevated brain natriuretic peptide and fibroblast growth factor 23 are associated with atrial fibrillation and improve detection of patients with atrial fibrillation

**Figure 3:**
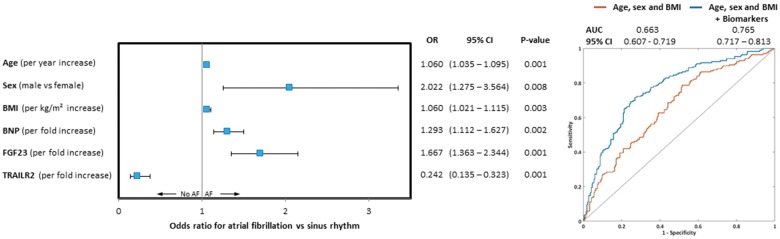
Odds ratios of the logistic regression model predicting atrial fibrillation (discovery cohort). Three clinical risk factors (age, sex, and body mass index) and two biomarkers (brain natriuretic peptide and fibroblast growth factor 23) were associated with increased odds of having atrial fibrillation, whereas biomarker TNF-related apoptosis-induced ligand-receptor 2 was associated with decreased odds of having atrial fibrillation. No significant interaction between age and sex were found. Error bars represent the 95% confidence interval. BMI, body mass index; BNP, brain natriuretic peptide; FGF-23, fibroblast growth factor 23; TRAIL-R2, TNF-related apoptosis-induced ligand-receptor 2.

There was no significant difference in sex distribution, BMI, prior stroke, hypertension, and heart failure between the groups (*Table 1*). Patients with AF were older than patients without AF, and were less likely to have diabetes or coronary artery disease. Medications also differed as expected in patients with AF, with higher use of oral anticoagulants, rate and rhythm control drugs than in patients without AF. The forward selection process identified three clinical risk factors to be significantly associated with AF: male sex, odds ratio (OR) 2.022 [95% confidence interval (CI) = 1.28–3.56, *P *=* *0.008] older age (OR = 1.060 per year increase; 95% CI = 1.04–1.10, *P *=* *0.001); and higher BMI (OR = 1.060 per BMI unit increase; 95% CI = 1.02–1.12, *P *=* *0.003; *Figure 3*).

**Table 1:** Baseline demographics of the study population

	Discovery		Validation	
	No AF (*n* = 215)	AF (*n* = 169)	No AF (*n* = 129)	AF (*n* = 125)
Age (years)^a^	66.0 (57.0–74.0)	73.0 (63.0–79.0)^b^	67.0 (59.1–74.0)	75.0 (67.0–81.5)^b^
Male	130 (60.5)	117 (69.2)	83.0 (64.3)	68.0 (54.4)
Ethnicity				
Caucasian	133.0 (61.9)	142.0 (84.0)^b^	104.0 (80.6)	116.0 (92.8)^b^
Asian	55.0 (25.6)	14.0 (8.3)	13.0 (10.1)	5.0 (4.0)
Afro-Caribbean	25.0 (11.6)	9.0 (5.3)	12.0 (9.3)	4.0 (3.2)
Unknown	2.0 (0.9)	4.0 (2.4)	—	—
BMI (kg/m^2^)^a^	28.1 (25.2–32.7)	29.6 (26.0–33.6)	29.1 (25.5–33.4)	28.9 (24.8–32.9)
eGFR (mL/min/1.73 m²)^a^	72.0 (57.0–87.0)	69.0 (57.5–84.0)	73.0 (58.3–85.0)	64.0 (44.5–79.0)^b^
Diabetes	89.0 (41.4)	37.0 (21.9)^b^	56.0 (43.4)	26.0 (20.8)^b^
Stroke	24.0 (11.2)	21.0 (12.4)	13.0 (10.1)	10.0 (8.0)
CAD	87.0 (40.5)	29.0 (17.2)^b^	78.0 (60.5)	29.0 (23.2)^b^
Hypertension	142.0 (66.0)	104.0 (61.5)	89.0 (69.0)	61.0 (48.8)
Heart failure	31.0 (14.4)	28.0 (16.6)	8.0 (6.2)	12.0 (9.6)
Ejection fraction (%)^a^	60.0 (53.1–67.3)	57.7 (45.0–65.0)^b^	57.0 (45.5–62.5)	55.0 (41.3–61.0)
Admission criteria				
Inpatient	160 (41.6)	97 (25.3)	124 (48.8)	97 (38.2)
Outpatient	55 (14.3)	72 (18.8)	5 (2.0)	28 (11.0)
Concomitant medication				
NOAC	4.0 (1.9)	63.0 (37.3)^b^	1.0 (0.8)	44.0 (35.2)^b^
VKA	5.0 (2.3)	48.0 (28.4)^b^	2.0 (1.6)	41.0 (32.8)^b^
Aspirin	137.0 (63.7)	39.0 (23.1)^b^	98.0 (76.0)	41.0 (32.8)^b^
Antiplatelet agents (clopidogrel, prasugrel, and ticagrelor)	94.0 (43.7)	33.0 (19.5)^b^	82.0 (63.6)	27.0 (21.6)^b^
ACEi	44.0 (20.5)	36.0 (21.3)	58.0 (45.0)	37.0 (29.6)^b^
Angiotensin II receptor blocker	39.0 (18.1)	28.0 (16.6)	22.0 (17.1)	25.0 (20.0)
Beta-blocker	115.0 (53.5)	83.0 (49.1)	85.0 (65.9)	72.0 (57.6)
Diuretic	59.0 (27.4)	66.0 (39.1)^b^	37.0 (28.7)	55.0 (44.0)^b^
Calcium channel antagonist	61.0 (28.4)	42.0 (24.9)	39.0 (30.2)	24.0 (19.2)^b^
Cardiac glycoside	—	33.0 (19.5)^b^	—	28.0 (22.4)^b^
Aldosterone antagonist	13.0 (6.0)	12.0 (7.1)	5.0 (3.9)	10.0 (8.0)
Verapamil/diltiazem	12.0 (5.6)	14.0 (8.3)	5.0 (43.9)	7.0 (5.6)
Antiarrhythmics (amiodarone, dronedarone, flecainide, propafenone, and sotalol)	4.0 (1.9)	17.0 (10.1)^b^	3.0 (2.3)	12.0 (9.6)^b^

Categorical variables are reported as *n* (%), whereas continuous variables are reported as mean (standard deviation) [or median (IQR) for non-parametric distributions]. The independent *t*-test (or Mann–Whitney *U* test for non-parametric distributions) and χ^2^ tests were used to compare continuous and categorical characteristics between patients within the two cohorts.

ACEi, angiotensin-converting enzyme inhibitor; BMI, body mass index; CAD, coronary artery disease; eGFR, estimated glomerular filtration rate; NOAC, non-vitamin K antagonist oral anticoagulant; VKA, vitamin K antagonist.

a Non-parametric distributions.

b A two-tailed significant difference *P* < 0.05 between patients with and without AF.

**Figure 4:**
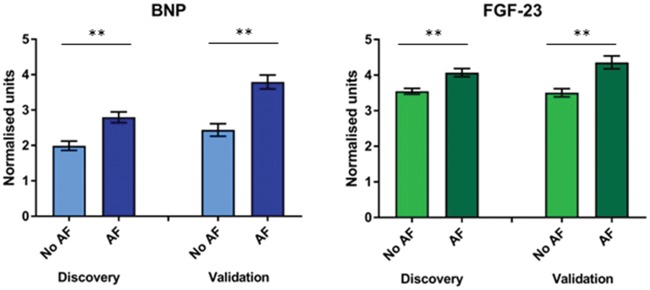
Comparison of biomarker levels between patients with and without atrial fibrillation. Elevated brain natriuretic peptide and fibroblast growth factor 23 levels observed in atrial fibrillation groups in both discovery and validation cohorts. **P < 0.001; error bars represent the SEM. BNP, brain natriuretic peptide; FGF-23, fibroblast growth factor 23.

Three biomarkers were also selected: elevated BNP and fibroblast growth factor-23 (FGF-23) were robustly associated with AF (OR = 1.293 per fold change increase; 95% CI = 1.11–1.63; *P *=* *0.002 and OR = 1.667, 95% CI = 1.36–2.34; *P *=* *0.001, respectively). Both BNP {*U *=* *13517, *P *<* *0.001; median [interquartile range (IQR)] 1.650 (0.522–3.917) vs. 2.958 (01.458–4.589)} and FGF-23 [*U *=* *14263, *P *<* *0.001; median (IQR) 3.330 (2.784–3.984) vs. 3.604 (3.067–4.946)] were significantly elevated in patients with AF compared with those in sinus rhythm (*Figure 4*). The increase in BNP and FGF-23 was not correlated with duration of AF (BNP, *P*=0.879; FGF-23, *P*=0.932; Supplementary material online, *Analysis A2*). In contrast, reduced TRAIL-R2 levels were associated with AF (OR = 0.242 per fold change increase, 95% CI = 0.14–0.32, *P *=* *0.001), but TRAIL-R2 concentrations were not different between patients in AF and in sinus rhythm (*P *=* *0.727). This model of three clinical risk factors and three biomarkers has an AUC of 0.765 (95% CI 0.717–0.813, *Table 2*) and a Brier score 0.197, with comparable values in the validation cohort [AUC 0.684 (95% CI = 0.62–0.75), Brier score 0.232].

### Validation by machine learning and sensitivity analyses

**Figure 5:**
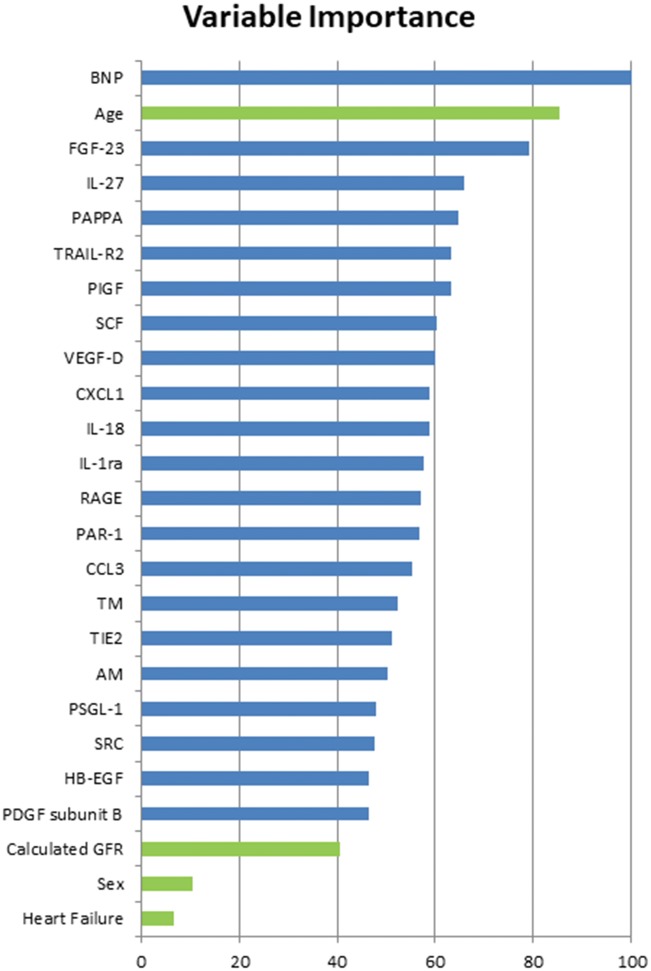
Feature selection using Random Forest. Seven clinical risk factors and 40 biomarkers were initially considered for inclusion. Backward selection with Random Forest was used to identify the model with the best area under the receiver-operator curve. Twenty-five variables were selected in the best model (four clinical risk factors in green; 21 biomarkers in blue) and ranked by importance with the most important variable given a score of 100. AM, adrenomedullin; BNP, brain natriuretic peptide; CCL3, C-C motif chemokine 3; CXCL1, C-X-C motif chemokine 1; FGF-23, fibroblast growth factor 23; HB-EGF, heparin-binding EGF-like growth factor; IL-18, interleukin-18; IL-1ra, interleukin-1 receptor antagonist protein; IL-27, interleukin-27; PAPPA, pappalysin-1; PAR-1, proteinase-activated receptor 1; PDGF subunit B, Platelet-derived growth factor subunit B; PIGF, placenta growth factor; PSGL-1, P-selectin glycoprotein ligand 1; RAGE, receptor for advanced glycosylation end products; SCF, stem cell factor; SRC, proto-oncogene tyrosine-protein kinase Src; TIE2, angiopoietin-1 receptor; TM, thrombomodulin; TRAIL-R2, TNF-related apoptosis-induced ligand-receptor 2; VEGF-D, vascular endothelial growth factor D.

As a complementary approach to account for dataset complexity and also to broaden the rubric of the statistical model, we performed machine learning analyses on the data. The process of feature selection identified a subset of variables that were most relevant for building the model. The variables selected, in decreasing order of importance, were BNP, age, FGF-23, IL-27, PAPPA, TRAIL-R2, PIGF, SCF, VEGF-D, CXCL1, IL-18, IL-1ra, RAGE, PAR-1, CCL3, TM, TIE2, ADM, PSGL-1, SRC, HB-EGF, PDGF subunit B, eGFR, sex, and heart failure (*Figure 5*). The best cross-validation algorithm was the Lasso and elastic-net regularized generalized linear model with an AUC of 0.697 (95% CI = 0.63–0.76). The algorithms ranked variables by order of scaled importance (SI) according to the most important variable (ranked as 1; *Table 3*). There were considerable overlaps in important variables ranked by the algorithms with the variables identified using forward selection. Particularly, age and biomarkers associated with AF identified in the logistic regression were highly ranked.

**Take home figure:**
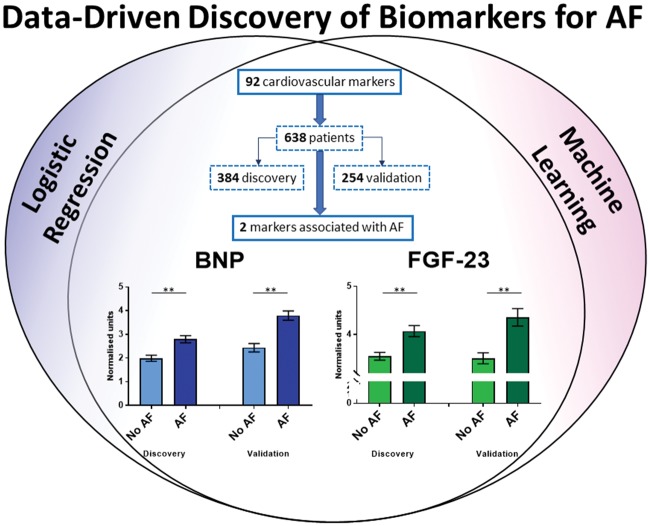
Data-driven discovery identifies BNP and FGF-23 as biomarkers for AF. Brain natriuretic peptide and fibroblast growth factor 23 identified by regression and machine learning to be robustly associated with atrial fibrillation in a cohort of 638 patients presenting to hospital. AF, atrial fibrillation; BNP, brain natriuretic peptide; FGF-23: fibroblast growth factor 23.

**Table 2:** Comparison of models predicting atrial fibrillation

Model	AUC	95% CI		Brier score
		Lower	Upper	
Age only (years)	0.618	0.562	0.675	0.238
Clinical risk factors only (age, sex, and BMI)	0.659	0.605	0.713	0.216
Clinical risk factors and biomarkers (age, sex, BMI, BNP, FGF-23, and TRAIL-R2)	0.765	0.717	0.813	0.197

Bootstrapped performance measures between three models showing incremental improvement with the addition of clinical risk factors and biomarkers identified by the logistic regression.

AUC, area under the ROC curve; BMI, body mass index; BNP, brain natriuretic peptide; FGF-23, fibroblast growth factor 23; TRAIL-R2, TNF-related apoptosis-induced ligand receptor 2.

In our sensitivity analysis, when logistic regression with forward selection was applied to all 92 biomarkers in the first 450 patients (discovery cohort), elevated NT-proBNP (precursor fragment of BNP; OR = 1.918 per fold change increase; 95% CI = 1.51–2.94; *P *=* *0.001), and FGF-23 (OR = 1.721 per fold change increase; 95% CI = 1.43–2.45; *P *=* *0.001) were associated with high odds of having AF. In addition, three other biomarkers were also identified [monocyte chemotactic protein 1, MCP-1 (OR = 1.799; 95% CI = 1.32–3.49, *P *=* *0.003), ST2 (OR = 1.607; 95% CI = 1.12–2.33, *P *=* *0.003), and receptor for advanced glycation end products, RAGE (OR = 1.656; 95% CI = 1.11–2.88, *P *=* *0.012)].

### Effect of biomarkers and imaging on atrial fibrillation prediction

To assess the clinical usefulness of biomarkers for identifying patients with AF, we compared the models using only age, only clinical risk factors, and the model including both clinical risk factors and biomarkers. The model with clinical risk factors performed better than age alone, and the addition of biomarkers to the clinical risk factors resulted in a significant net gain in reclassification of 11.2% (35 correctly reclassified, 16 incorrectly reclassified, *P *=* *0.008) for patients who had AF and 6.5% for patients without AF (28 correctly reclassified, 14 incorrectly reclassified, *P *=* *0.031), yielding an overall NRI of 0.178 (*P *<* *0.001; *Table 3*). The AUC and Brier score improved as well.

**Table 3:** Ranking of the 10 most important variables for each of the algorithms run

Algorithms Ranking	Lasso and elastic-net regularized generalized linear model	Support vector machines with linear Kernel	Random forest	Stochastic gradient boosting	Recursive partitioning
1	**Age**	**BNP**	**Age**	PIGF	PSGL-1
2	**TRAIL-R2**	**Sex**	**FGF-23**	**FGF-23**	**FGF-23**
3	RAGE	**FGF-23**	IL-27	SCF	CXC1
4	TM	VEGF-D	**BNP**	PAPPA	TIE2
5	PAR-1	IL-27	PDGF sub-B	VEGF-D	**Age**
6	VEGF-D	RAGE	SRC	IL-27	IL-27
7	IL-1ra	CCL3	**TRAIL-R2**	**Age**	PAPPA
8	PAPPA	ADM	ADM	**TRAIL-R2**	RAGE
9	PSGL-1	SCF	**Sex**	PSGL-1	TM
10	**Sex**	PAPPA	IL-1ra	**BNP**	IL-1ra

The most important variable is ranked as 1. Note that collinearity exists in machine learning techniques, allowing the best set of related variables to determine predictive accuracy. Variables which overlap with the forward selection logistic regression are in bold.

ADM, adrenomedullin; BNP, brain natriuretic peptide; CCL3, C-C motif chemokine 3; CXCL1, C-X-C motif chemokine 1; FGF-23, fibroblast growth factor 23; IL-1ra, interleukin-1 receptor antagonist protein; IL-27, interleukin-27; PAPPA, pappalysin-1; PAR-1, proteinase-activated receptor 1; PDGF subunit B, platelet-derived growth factor subunit B; PIGF, placenta growth factor; PSGL-1, P-selectin glycoprotein ligand 1; RAGE, receptor for advanced glycosylation end products; SCF, stem cell factor; SRC, Proto-oncogene tyrosine-protein kinase Src; TIE2, angiopoietin-1 receptor; TM, thrombomodulin; TRAIL-R2, TNF-related apoptosis-induced ligand-receptor 2; VEGF-D, vascular endothelial growth factor D.

When echocardiography parameters were included in the forward selection, left atrial dilation was selected, whereas BMI and BNP were dropped from the model; both FGF-23 and TRAIL-R2 remained in the model. Subsequent model fitting indicated a significant association between left atrial dilation and increased odds for AF (OR = 2.809, 95% CI = 1.70–5.24; *P *=* *0.001). We found marked BNP elevation in patients with atrial dilation [*U *=* *6165, *P *<* *0.001; median (IQR) 3.441 (1.797–5.293) vs. 1.006 (0.482–2.839) without left atrial dilation].

## Discussion

This data-driven assessment of common cardiovascular biomarkers confirmed prior reports that AF was associated with elevated BNP levels.^10^^,^^20^ We also identified FGF-23—a protein associated with cardiac hypertrophy, chronic kidney disease, and vascular stiffness—as a robust marker for AF.^21^^,^^22^ A simple assessment of age, sex, BMI, and these two biomarkers robustly identified patients with AF in both the discovery and validation cohorts in our study.

### Clinical implications

Including biomarker measurements in clinical practice could better identify patients with undiagnosed prevalent AF. A point-of-care test for BNP and/or FGF-23 could allow such screening in many settings, especially in environments without immediate input from medically trained personnel.^7^^,^^23^ This can refine ongoing approaches using only age and BNP to select patients for screening.^23^

The detailed phenotyping in our study allowed transthoracic echocardiography imaging parameters to be integrated in our analyses. Brain natriuretic peptide was elevated in patients with dilated left atrium, rendering BNP a potential marker for atrial dilation *in lieu* of imaging. Thus, a BNP test can facilitate screening for AF in settings without cardiac imaging, e.g. in community and primary care. Conversely, BNP measurements could be omitted in patients undergoing cardiac imaging with quantification of left atrial size. Further validation is needed to confirm this proposition.

### Implications for stratified prevention and therapy

Our data confirmed elevated BNP (and its precursor fragment NT-proBNP)^10^^,^^14^ as a marker for AF and for atrial dilation. Brain natriuretic peptide is a natriuretic peptide synthesized by cardiomyocytes in response to increased pressure and myocardial stretch.^24^ Our findings illustrate the importance of detecting increased atrial load and strain for identifying AF in patients. Load reduction—e.g. through antihypertensive therapy—which appears to have potential for prevention of AF,^25^ could be particularly useful to treat AF in patients with atrial dilation or elevated BNP.

FGF-23 is elevated with decreasing kidney function and is associated with all-cause mortality and cardiovascular disease in patients with chronic kidney disease.^26^ The relationship between elevated FGF-23 levels with AF and left ventricular function, independently of kidney disease, was unclear until now in the literature, showing association in some cohorts,^21^ but not in others.^27^ Our study confirms that FGF-23 levels are elevated in patients with AF.

FGF-23 is a phosphate and calcium-regulating hormone primarily secreted by osteocytes and osteoblasts. As FGF-23 promotes myocardial remodelling and cardiac hypertrophy,^28^ it can cause or enhance hypertrophy-related ectopic activity and automaticity, leading to AF. FGF-23 is also associated with endothelial dysfunction.^29^ It is possible that all of the mechanisms discussed contribute in some part to the development of AF in patients with elevated FGF-23.^22^^,^^28^^,^^29^

We observed an inverse association of TRAIL-R2 with AF, but we did not find a difference in TRAIL-R2 concentrations between patients in sinus rhythm and in AF. This observation most likely reflects complex interactions between AF and clinical characteristics in our patient demographics which show an enrichment of patients with coronary artery disease and diabetes in sinus rhythm.

In summary, our analysis suggests that volume load (reflected by elevated BNP) and cardiac stiffness (reflected by elevated FGF-23) are two major drivers of AF, possibly pointing to two clinically relevant types of AF.^30^ Clearly, further research is warranted to understand the mechanisms linking elevated FGF-23 and BNP to AF.

### Limitations

Supporting our findings, both conventional statistical techniques and novel machine learning analyses yielded similar results, but there are limitations. Firstly, we acknowledge potential observation biases for patient selection, although our inclusion criteria were broad, creating a data set that is representative of the range of patients referred to hospital. In addition, there was a small number of missing data which were imputed. Statistical approaches were applied to minimise over-fitting, and a variety of analytical approaches supported our main results, but our findings, particularly pertaining to FGF-23, need further external validation in separate populations.

Conceptually, biomarker measurements in a population-based sample and long-term follow-up for incident AF are desirable for validation of our findings. Fortunately, longitudinal population-based studies for incident AF^9^^,^^15^ have identified similar markers for AF as cross-sectional studies (mainly BNP), suggesting that markers for prevalent AF also identify patients at risk of incident AF.

## Conclusions

Elevated BNP is an established marker for prevalent AF, while elevated FGF-23 as a new biomarker robustly associated with AF. A simple assessment of age, sex, BMI, BNP, and FGF-23 can identify patients with AF, e.g. to enrich populations undergoing ECG screening. Brain natriuretic peptide and FGF-23 may also be useful to stratify patients with AF.

## Supplementary Material

Supplementary Material
