# Supplementary material for: Data-driven discovery and validation of circulating blood-based biomarkers associated with prevalent atrial fibrillation
Source: Eur Heart J. 2019 Jan 7;40(16):1268–76. doi: 10.1093/eurheartj/ehy815 (PMC6475521; doi:10.1093/eurheartj/ehy815)
Supplement: Supplementary Material [file ehy815_supplementary_material.docx]

*Supplementary Table S1: Inclusion and exclusion criteria of the BBC-AF registry.*

| **Inclusion criteria** |
| --- |
| *AF diagnosed by ECG*  ***or*** |
| *One of the following:* |
| - Age ≥ 75 years old |
| - Prior stroke / TIA |
|  |
| *Or two of the following:* |
| - Age ≥ 65 years old |
| - Female |
| - Hypertension (on chronic treatment for hypertension / on antihypertensive treatment / resting blood pressure > 145/90 mmHg) |
| - Diabetes mellitus or impaired glucose tolerance |
| - Severe coronary artery disease, CAD (MI past or present, CABG or PCI) |
| - Stable heart failure (NYHA class II or above; or left ventricular ejection fraction < 50%) |
| - Left ventricular hypertrophy on echocardiography (left ventricular posterior wall / interventricular septal thickness ≥ 12 mm) |
| - Peripheral artery disease |
|  |
| ***And:*** |
| - Able to provide informed consent |
| - Age ≥ 18 years old |
|  |
| **Exclusion criteria** |
| - Unable / unwilling for follow up |
| - Unwillingness to undergo investigations required (e.g. echocardiography / event recorders). |
| - Life expectancy at recruitment < 1 year |

*AF: Atrial fibrillation, TIA: Transient ischaemic attack, CAD: Coronary artery disease, MI: Myocardial infarction, CABG: Coronary artery bypass graft, PCI: Percutaneous coronary intervention, NYHA: New York Heart Association.*

*Supplementary Table S2: List of 40 biomarkers analysed for both discovery and validation cohorts which were quantified using the Olink CVD I and CVD II panels.*

| **Biomarker** | **Abbreviation** |
| --- | --- |
| Adrenomedullin | ADM |
| Agouti-related protein | AGRP |
| Angiopoietin-1 receptor | TIE2 |
| Cathepsin L1 | CTSL1 |
| C-C motif chemokine 3 | CCL3 |
| CD40 ligand | CD40L |
| C-X-C motif chemokine 1 | CXCL1 |
| Dickkopf-related protein 1 | Dkk-1 |
| Fibroblast growth factor 23 | FGF-23 |
| Follistatin | FS |
| Growth hormone | GH |
| Heat shock 27 kDa protein | HSP 27 |
| Heparin-binding EGF-like growth factor | HB-EGF |
| Interleukin-1 receptor antagonist protein | IL-1ra |
| Interleukin-16 | IL-16 |
| Interleukin-18 | IL-18 |
| Interleukin-27 | IL-27 |
| Interleukin-6 | IL-6 |
| Lectin-like oxidized LDL receptor | LOX-1 |
| Leptin | LEP |
| Matrix metalloproteinase-12 | MMP-12 |
| Matrix metalloproteinase-7 | MMP-7 |
| Melusin | ITGB1BP2 |
| Natriuretic peptides B | BNP |
| NF-kappa-B essential modulator | NEMO |
| Pappalysin-1 | PAPPA |
| Pentraxin-related protein PTX3 | PTX3 |
| Placenta growth factor | PIGF |
| Platelet-derived growth factor subunit B | PDGF subunit B |
| Proteinase-activated receptor 1 | PAR-1 |
| Proto-oncogene tyrosine-protein kinase Src | SRC |
| P-selectin glycoprotein ligand 1 | PSGL-1 |
| Receptor for advanced glycosylation end products | RAGE |
| Renin | REN |
| Stem cell factor | SCF |
| Thrombomodulin | TM |
| TIM.1 | TIM-1 |
| Tissue factor | TF |
| TNF-related apoptosis-induced ligand receptor 2 | TRAIL-R2 |
| Vascular endothelial growth factor D | VEGF-D |

*Supplementary Table S3: List of 92 biomarkers included in the sensitivity analysis.*

| **Biomarker** | **Abbreviation** |
| --- | --- |
| Adrenomedullin | AM |
| Agouti-related protein | AGRP |
| Angiopoietin-1 receptor | TIE2 |
| Beta-nerve growth factor | Beta-NGF |
| Caspase-8 | CASP-8 |
| Cathepsin D | CTSD |
| Cathepsin L1 | CTSL1 |
| C-C motif chemokine 3 | CCL3 |
| C-C motif chemokine 4 | CCL4 |
| C-C motif chemokine 20 | CCL20 |
| CD40 ligand | CD40L |
| Chitinase-3-like protein 1 | CXCL6 |
| C-X-C motif chemokine 1 | CXCL1 |
| C-X-C motif chemokine 6 | CXCL6 |
| C-X-C motif chemokine 16 | CXCL16 |
| Cystatin-B | CSTB |
| Dickkopf-related protein 1 | Dkk-1 |
| Endothelial cell-specific molecule 1 | ESM-1 |
| Eosinophil cationic protein | ECP |
| Epidermal growth factor | EGF |
| E-selectin | SELE |
| Fatty acid-binding protein, adipocyte | FABP4 |
| Fibroblast growth factor 23 | FGF-23 |
| Follistatin | FS |
| Fractalkine | CX3CL1 |
| Galanin peptides | GAL |
| Growth differentiation factor 15 | GDF-15 |
| Growth hormone | GH |
| Heat shock 27 kDa protein | HSP 27 |
| Heparin-binding EGF-like growth factor | HB-EGF |
| Hepatocyte growth factor | HGF |
| Interleukin-1 receptor antagonist protein | IL-1ra |
| Interleukin-4 | IL-4 |
| Interleukin-6 | IL-6 |
| Interleukin-6 receptor subunit alpha | IL-6RA |
| Interleukin-8 | IL-8 |
| Interleukin-16 | IL-16 |
| Interleukin-18 | IL-18 |
| Interleukin-27 | IL-27 |
| Kallikrein-6 | KLK6 |
| Kallikrein-11 | hk11 |
| Lectin-like oxidized LDL receptor | LOX-1 |
| Leptin | LEP |
| Macrophage colony-stimulating factor 1 | CSF-1 |
| Matrix metalloproteinase-1 | MMP-1 |
| Matrix metalloproteinase-3 | MMP-3 |
| Matrix metalloproteinase-10 | MMP-10 |
| Matrix metalloproteinase-12 | MMP-12 |
| Matrix metalloproteinase-7 | MMP-7 |
| Melusin | ITGB1BP2 |
| Membrane-bound aminopeptidase P | mAmP |
| Monocyte chemotactic protein 1 | MCP |
| Myeloperoxidase | MPO |
| Myoglobin | MB |
| Natriuretic peptides B | BNP |
| NF-kappa-B essential modulator | NEMO |
| N-terminal pro-B-type natriuretic peptide | NT-pro-BNP |
| Osteoprotegerin | OPG |
| Ovarian cancer-related tumor marker 125 | CA-125 |
| Pappalysin-1 | PAPPA |
| Pentraxin-related protein PTX3 | PTX3 |
| Placenta growth factor | PIGF |
| Platelet endothelial celll adhesion molecule | PECAM-1 |
| Platelet-derived growth factor subunit B | PDGF subunit B |
| Prolactin | PRL |
| Protein S100-A | EN-RAGE |
| Proteinase-activated receptor 1 | PAR-1 |
| Proto-oncogene tyrosine-protein kinase Src | SRC |
| P-selectin glycoprotein ligand 1 | PSGL-1 |
| Receptor for advanced glycosylation end products | RAGE |
| Renin | REN |
| Resistin | RETN |
| SIR2-like protein | SIRT2 |
| Spondin-1 | SPON1 |
| ST2 protein | ST2 |
| Stem cell factor | SCF |
| Thrombomodulin | TM |
| TIM.1 | TIM-1 |
| Tissue factor | TF |
| Tissue-type plasminogen activator | t-PA |
| TNF-related activation-induced cytokine | TRANCE |
| TNF-related apoptosis-inducing ligand | TRAIL |
| Tumour necrosis factor ligand superfamily member 14 | TNFSF14 |
| Tumour necrosis factor receptor 1 | TNF-R1 |
| Tumour necrosis factor receptor 2 | TNF-R2 |
| TNF-related apoptosis-induced ligand receptor 2 | TRAIL-R2 |
| Tumour necrosis factor receptor superfamily member 5 | CD40 |
| Tumour necrosis factor receptor superfamily member 6 | FAS |
| Urokinase plasminogen activator surface receptor | U-PAR |
| Vascular endothelial growth factor A | VEGF-A |
| Vascular endothelial growth factor D | VEGF-D |

***Supplementary methods***

*Biomarker quantification*

The blood samples of all patients were analysed using a validated proximity extension assay (PEA) platform which simultaneously measures 92 proteins related to cardiovascular conditions (Olink Proteomics, Uppsala, Sweden). The samples were prepared with incubations which determined the lower detection limit (LOD) and for normalising the measurements. The LOD referred to the lowest measurable level of an individual protein and was defined as three times the standard deviation over background levels determined by the negative control. Samples were flagged if the control sample deviated more than ± 0.3 from the median value of all samples on the particular run. Ninety samples were measured on each run with three interpolate controls and three negative controls; eight runs were used for quantification of all samples. Protein expression was quantified and set relative to a correction factor to generate a Normalised Protein eXpression unit (NPX) - higher NPX values therefore represent higher protein levels with 1 NPX difference equaling a two-fold increase in protein concentration. Values below the detection limit of the assay were replaced by the lower limit of detection. Batch effects were corrected using an empirical Bayes method (ComBat)[^1^](#_ENREF_1). The final dataset for modelling contained 10 missing biomarker data points (Cathepsin L1 = 3; Follistatin= 1; Pentraxin-related protein PTX3 = 3; P-selectin glycoprotein ligand 1 = 2; Thrombomodulin = 1) which were imputed using Multivariate Imputation by Chained Equations. Missing values for clinical characteristics were also imputed (N, % missing): BMI (18, 4.7%), eGFR (70, 18.2%), Mitral regurgitation (58, 15.1%), Left atrial dilation (62, 16.1%).

*Machine learning*

The R language for statistical computing [^2^](#_ENREF_2) was used with the ROSE [^3^](#_ENREF_3), caret [^4^](#_ENREF_4), randomForest [^5^](#_ENREF_5)¸ e1071 [^6^](#_ENREF_6), glmnet [^7^](#_ENREF_7), rpart [^7^](#_ENREF_7), gbm [^8^](#_ENREF_8) and pROC [^9^](#_ENREF_9) packages. The variable importance for the random forest feature selection was calculated using the classification error for the random forest trees and the error after permuting the predictor variables [^5^](#_ENREF_5). Models with the selected features were trained using Random Forest [^5^](#_ENREF_5), Support Vector Machines with Linear Kernel [^6^](#_ENREF_6), Lasso and Elastic-Net Regularized Generalized Linear Model [^7^](#_ENREF_7), Recursive Partitioning [^10^](#_ENREF_10) and Stochastic Gradient Boosting [^8^](#_ENREF_8). For the final model selected, (Lasso and Elastic-Net Regularized Generalized Linear Model), the variable importance was calculated using the scaled absolute value of the coefficients of the model [^7^](#_ENREF_7). Other algorithms use the same method as described in the caret package [^4^](#_ENREF_4).

***Supplementary Analysis***

*A1: Relation between biomarker levels and duration of AF*

Duration of AF was calculated at time of recruitment and regressed against BNP and FGF-23 biomarker levels for all patients diagnosed with AF who have this information available (N=179 of 297 patients with AF). There was no significant correlation between duration of AF and biomarker levels (BNP *p* = .879; FGF-23 *p* = .932). The results indicate no relationship between duration of AF and degree of elevation of BNP and FGF-23 levels.

***Supplementary figure F1:*** *Lack of correlation of biomarker levels with duration of AF.*

***
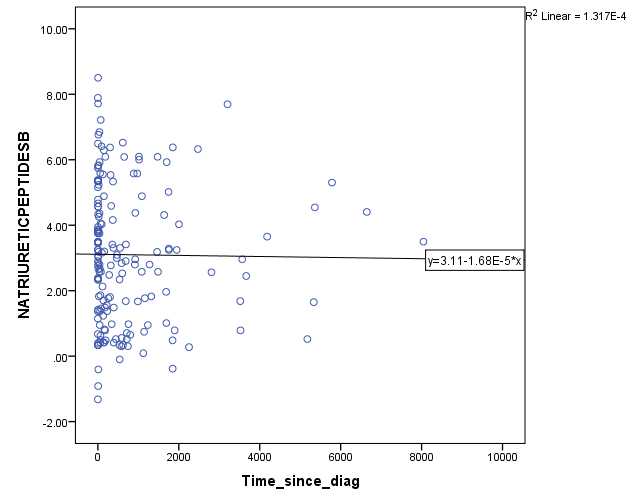

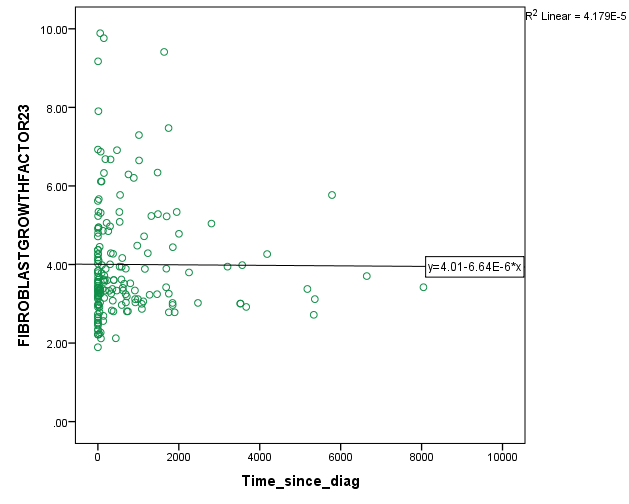
***

*Levels of BNP (left panel, in blue) and FGF-23 (right panel, in green) in patients diagnosed with AF showed non-significant correlations with duration of AF (in days).*

*A2: Random case sampling*

Random sampling was performed to select cases for the discovery and validation cohorts according to the 60:40 ratio. The selection for logistic regression was done in SPSS and for machine learning using R. For each of the five runs performed, the seed for the random number generator was reset.

***Supplementary Table S4:*** *Outcome of the forward selection process for five runs of random case sampling, with their respective AUCs for the discovery and validation cohorts.*

|  | **Run 1** | **Run 2** | **Run 3** | **Run 4** | **Run 5** |
| --- | --- | --- | --- | --- | --- |
|  | **BNP** | Leptin | **BNP** | **BNP** | Leptin |
|  | RAGE | **BNP** | HB-EGF | MMP-12 | **BNP** |
|  | **FGF-23** | HB-EGF | **FGF-23** | HB-EGF | HB-EGF |
|  | **TRAIL-R2** | **FGF-23** | **TRAIL-R2** | **FGF-23** | **FGF-23** |
|  | IL-27 | **TRAIL-R2** | PDGF-B | **TRAIL-R2** | **TRAIL-R2** |
|  | **Age** | IL-27 | CD40-L | PAR-1 | IL-27 |
|  | **BMI** | CD40-L | **Age** | **Age** | PAR-1 |
|  |  | **Age** | **BMI** | HTN | **Age** |
|  |  | **BMI** | **Sex** |  | **BMI** |
|  |  | HTN | HTN |  | **Sex** |
| **AUC** |  |  |  |  | HTN |
| Discovery | .734 | .796 | .795 | .782 | .801 |
| Validation | .773 | .745 | .740 | .715 | .750 |

*Variables that overlap with the current forward selection logistic regression model are in bold. All three biomarkers identified using the case selection by biomarker panel (BNP, FGF-23, TRAIL-R2) were selected in all five runs using random case selection. BNP: Brain natriuretic peptide, RAGE: Receptor for advanced glycosylation end products,* *FGF-23: Fibroblast growth factor 23, TRAIL-R2: TNF-related apoptosis-induced ligand receptor 2, IL-27: Interleukin-27, HB-EGF: Heparin-binding EGF-like growth factor, CD40-L: CD40 ligand, PDGF-B: Platelet-derived growth factor subunit B, MMP-12: Matrix metalloproteinase-12, PAR-1: Proteinase-activated receptor 1, BMI: Body mass index, HTN: Hypertension.*

***Supplementary Table S5:*** *Outcome of the machine learning ranking by scaled importance for five runs of random case sampling.*

|  | **Run 1** | | **Run 2** | | **Run 3** | | **Run 4** | | **Run 5** | |
| --- | --- | --- | --- | --- | --- | --- | --- | --- | --- | --- |
|  | **5/5** | **4/5** | **5/5** | **4/5** | **5/5** | **4/5** | **5/5** | **4/5** | **5/5** | **4/5** |
|  | **FGF-23** | RAGE | **BNP** | RAGE | **Age** | RAGE | **FGF-23** | **BNP** | **BNP** | **FGF-23** |
|  | HB-EGF | ADM | **FGF-23** |  | **FGF-23** | PAPPA | GH | **Age** | **Age** |  |
|  | **BNP** | PIGF | **Age** |  | **BNP** | CD40-L |  | IL-27 | IL-27 |  |
|  | **Age** | TF | IL-27 |  | GH | PIGF |  | RAGE | TF |  |
|  | CCL3 | PAPPA | GH |  | IL-27 |  |  | TF | GH |  |
|  |  |  |  |  | NEMO |  |  | CCL3 |  |  |
|  |  |  |  |  | eGFR |  |  |  |  |  |
| **Discovery** | **SVMLin2** | .688 | **SVMLin2** | .714 | **SVMLin2** | .687 | **Glmnet** | .696 | **SVMLin2** | .657 |
| **Validation** |  | .662 |  | .614 |  | .653 |  | .635 |  | .660 |

*For each run, five algorithms were applied. The variables which were selected by all five, and four out of five algorithms, as well as the respective AUCs for the best algorithm of each run, are listed. Variables that overlap with the current forward selection logistic regression model are in bold. FGF-23: Fibroblast growth factor 23, HB-EGF: Heparin-binding EGF-like growth factor, BNP: Brain natriuretic peptide, CCL3: C-C motif chemokine 3,* *RAGE: Receptor for advanced glycosylation end products,* ADM: *Adrenomedullin, PIGF: Placenta growth factor, TF: Tissue factor, PAPPA: Pappalysin-1, IL-27: Interleukin-27, GH: Growth hormone, NEMO: NF-kappa-B essential modulator, eGFR: estimated glomerular filtration rate, CD40-L: CD40 ligand, RF: Random forest, Glmnet: Lasso and Elastic-Net Regularized Generalized Linear Model, SVMLin2: Support Vector Machines with Linear Kernel.*

***References***

1. Johnson WE, Li C, Rabinovic A. Adjusting batch effects in microarray expression data using empirical Bayes methods. Biostatistics 2007;**8**(1):118-27.

2. R Core Team. R: A language and environment for statistical computing. In. Vienna, Austria: R Foundation for Statistical Computing; 2017.

3. Lunardon N, Menardi G, Torelli N. ROSE: A package for binary imbalanced learning. R Journal 2014;**6**(1):11.

4. Kuhn M, With contributions from Wing J, Weston S, Williams A, Keefer C, Engelhardt A, Cooper T, Mayer Z, Kenkel B, Team TRC, Benesty M, Lescarbeau R, Ziem A, Scrucca L, Tang Y, Candan C, Hunt T. caret: Classification and Regression Training. In. R package version 6.0-76 ed; 2017.

5. Liaw A, Wiener M. Classification and Regression by randomForest. R News 2002;**2**(3):5.

6. Meyer D, Dimitriadou E, Hornik K, Weingessel A, Leisch F. e1071: Misc Functions of the Department of Statistics. In. R package version 1.6-8 ed. TU Wien: Probability Theory Group (Formerly: E1071); 2017.

7. Friedman J, Hastie T, Tibshirani R. Regularization Paths for Generalized Linear Models via Coordinate Descent. Journal of Statistical Software 2010;**33**(1):22.

8. Ridgeway G, others cf. Generalized Boosted Regression Models. In. R package version 2.1.3 ed; 2017.

9. Robin X, Turck N, Hainard A, Tiberti N, Lisacek F, Sanchez JC, Muller M. pROC: an open-source package for R and S+ to analyze and compare ROC curves. BMC Bioinformatics 2011;**12**:77.

10. Therneau T, Atkinson B, Ripley B. Recursive Partitioning and Regression Trees. In. R package version 4.1-11. ed; 2017.
